# The trend of drowning before and after COVID-19 pandemic in Qazvin province: an area without sea shores in Iran 

**Published:** 2022-01

**Authors:** Leila Kouchakinejad-Eramsadati, Mostafa Amiri, Soheil Soltani, Kiyoumars Allahbakhshi

**Affiliations:** ^*a*^ Ph.D. Student in Health in Emergencies & Disasters, Department of Health in Emergencies & Disasters, School of Public Health, Tehran University of Medical Sciences, Tehran, Iran.; ^*b*^ BSN, Prehospital Medical Emergency Organization, Qazvin University of Medical Sciences, Qazvin, Iran.; ^*c*^ Medical Doctor, Emergency Medical Specialist, Qazvin University of Medical Sciences, Qazvin, Iran.; ^*d*^ Faculty Member, Department of Health in Emergencies & Disasters, School of Public Health, Tehran University of Medical Sciences, Tehran, Iran.

## Abstract

**Background:**

Drowning is one of the leading causes of unintentional mortality in Iran. The COVID-19 pandemic combined with drowning has indirectly affected the community. Qazvin province has had a remarkable rate of drowning. This study aimed to investigate the impact of COVID-19 on drowning in this province.

**Methods:**

In a cross-sectional study that used the national drowning registry database, all of the people in Qazvin who drowned between March 2017 to March 2021 were compared. Data were collected from the database of Emergency Medical Services in Qazvin and analyzed by SPSS software. Descriptive and Poisson regression and time series analysis were used.

**Results:**

The number of missions before the COVID-19 outbreak was 35.6 per year and 32 after it. The number of deaths increased from 12.3 to 15. The mean age of the patients was 27.53 and 26.92 years before and after the pandemic, respectively. May-June had the highest number of drownings. Most of the drownings before and after COVID-19 belonged to the male gender. The number of missions in rural and urban areas increased after the COVID-19 outbreak, while all drowning callings outside urban and rural areas decreased. According to Poisson's estimation regression model, the drowning trend diminished with a coefficient of -0.037 and was statistically significant at p = 0.032.

**Conclusions:**

The findings of the study indicated that COVID-19 did not impact the frequency pattern of drownings in Qazvin. However, the increased number of drownings in agricultural pools and urban water channels was significant. Travel restrictions, pool closures, and decreased global warming due to lockdown could have been effective on the number of drownings. Therefore, it is recommended that policymakers consider drowning prevention planning with priority given to young age groups.

**Keywords:**

COVID-19, Drowning, Qazvin Province

